# Book Reviews

**Published:** 1827-01

**Authors:** 


### CRITICAL ANALYSES.

Quæ laudanda forent, et quæ culpanda, vicissim

Illa, prius, cretâ; mox hæc, carbone, *notamus*.—PERSIUS.

*An Essay on Morbid Sensibility of the Stomach and Bowels, as the Proximate Cause, or Characteristic Condition, of Indigestion, Nervous Irritability, Mental Despondency, Hypochondriasis, &c. &c. To which are prefixed, Observations on the Diseases and Regimen of Invalids, on their Return from hot and unhealthy Climates.* By JAMES JOHNSON, M.D. of the Royal College of Physicians, &c.—8vo. pp. 128. Thos. and Geo. Underwood, London. 1827.

THIS Essay is a reprint of the additional matter in the fourth edition of Dr. JOHNSON's well-known and justly-esteemed work on the "Influence of Tropical Climates on European Constitutions," and is published separately for the convenience of those who may be possessed of a former edition, or who may not be inclined to possess the present one. To both the one and the other of these classes of persons we can conscientiously recommend this useful little work. It contains 128 closely printed pages, and as much matter as some authors, with the help of a large type and wide margin, would have expanded into a goodly octavo. The subject may be easily guessed: it is an old friend (or rather an old enemy) with a new face,—the dyspepsia and hypochondriasis of CULLEN,—the deranged digestive organs of ABERNETHY,—the bilious disorders of one author, and the stomach complaints of another. Dr. Johnson is dissatisfied with all and each of these terms, and he offers the following reasons for adopting a new one:

"When this combination of gastric and hepatic disorder obtains, whichever may have had the priority, the term 'indigestion' is merely a conventional one, which is meant to designate a complication in which indigestion forms at most but a part—a very small part—and sometimes no part at all. I own that it is very hard for any one but a German to give such a name to this complication as may convey a clear idea of its nature. By the term 'morbid sensibility of the stomach and bowels,' I mean a disordered condition of the gastric and intestinal nerves, in which their natural sensibility is changed, being morbidly acute, morbidly obtuse, (torpid) or perverted. By this term, I merely designate a fact or condition which, in my opinion, obtains much more generally in this class of maladies than the state called indigestion: indeed, I think I may aver that it is never absent in the functional disorders of the digestive apparatus now under review, and that it forms the connecting link between these disorders and the various sympathetic affections of other and distant parts of the system. This is my apology for the term." (P. 54.)

In a note, the author obviates, and we think very fairly, an objection to his newly-adopted term, which will at once occur to the reader.

"It may appear an incongruity to consider the organic sensibility of the stomach and bowels as morbidly increased at a time when the latter (the bowels) are generally supposed to be in a state of torpor, as evinced by constipation. But the *organic sensibility* of the bowels may be greatly perverted and exalted, and yet the muscular or peristaltic action irregular, or even torpid. Besides, it is a law of the animal economy, that, when nervous sensibility is too much excited in one part, it is too little so in some other. Thus, we often see the stomach and upper bowels in a state of great irritability, while the lower bowels are quite torpid, and will not propel forward their contents. Gastric irritability and vomiting are usually accompanied by constipation. Finally, I may observe, that the *functions* of the stomach, liver, and intestines, may be *torpid*, while the *organic* sensibility of their nerves may be in a state of morbid excitement." (P. 51.)

Previously, however, to entering upon the subject of morbid sensibility of the stomach and bowels, Dr. Johnson gives us forty pages on the "diseases and regimen of invalids returning from hot and unhealthy climates." We do not exactly see how this bears on the general questions discussed in this essay. The remarks, indeed, are well drawn up, and appear to us to form a valuable—we might say an indispensible—addition to any work professing to treat on the influence of tropical climates on European constitutions; but they have no direct reference to morbid sensibility of the intestinal canal considered abstractedly, and therefore we think them misplaced here. Had they been appended rather than prefixed to the main essay, the abruptness with which the work opens, and which every reader must be struck with, would at least have been avoided.

Among the diseases to which persons who have long resided in hot climates are exposed on their return to England, Dr. Johnson lays particular stress on those of the lungs and liver. Many of his observations on these affections, on their pathology especially and treatment, are sound and practically useful; but we could not forbear remarking a curious distinction which he draws in the diagnosis of diseases of these organs. In animadverting on the work of a contemporary, whose labours in our own department afford us at all times both instruction and example, we would avoid, of course, even the appearance of captious criticism; but there is something in the passage before us which calls for especial notice. We have never disguised our own opinions concerning the value of the French invention, the stethoscope;—Dr. Johnson, on the other hand, is one of its steadiest friends in this or any country. The very mantle of Laennec appears to have fallen upon his shoulders. Let us see, however, how much the possession of this instrument has warped his pathological opinions; and for this purpose let us compare his reasonings concerning the functional and organic diseases of the parts just named, the lungs and the liver.

Speaking of the propriety of distinguishing between the dyspeptic pulmonary affection, in which the mucous membrane is chiefly engaged, and that more advanced stage of pulmonary disorder in which the parenchymatous tissue of the lungs becomes involved, and when, if a scrofulous diathesis be present, tubercles are excited into action, he says—

"The grand object is to determine the period when *symptomatic disorder* is passing into the state of *actual disease;* and this, I maintain, cannot be done by any investigation of symptoms, however minute, short of exploration of the chest by means of auscultation and percussion. Yet, on this distinction between the two states, the whole question of treatment hinges." (P. 26.)

Further on, he uses these words—

"Without this investigation, then, we may be too early in our treatment of the pulmonic affection, or too late. The former error is dangerous, but the latter is fatal to the patient. If auscultation were attended with no other advantage than this discrimination of the two stages of dyspeptic phthisis, (a disease so very prevalent in this country,) it would be the most valuable discovery of the present century." (P. 27.)

Let the reader contrast these remarks with some that follow on the diagnosis of functional and organic disease of the liver.

"I have endeavoured to reduce the diagnosis within its proper, or at all events its practical, limits, and to restrain the vague notions respecting 'liver disease,' which are so prevalent and so detrimental. Indeed, I am convinced that, were the term and the idea of 'organic disease' of the liver obliterated, not only from the nosological chart, but from the minds of practitioners, it would be much better for their patients. No possible danger can accrue from mistaking an organic disease of the liver for a functional one, but much mischief may result from the contrary mistake. This will appear a strange position to be maintained, and is the reverse of that commonly laid down; but it is not stated without mature reflection. More diseases of structure in the liver would be cured by careful attention to its function, than by all the other means put together." (P. 40.)

Does it not follow from these premises, that, if an instrument should hereafter be discovered, by which the practitioner may be taught to distinguish between a tuberculated and a simply indurated state of liver, or any other chronically diseased condition of that viscus: in short, if an abdominal stethoscope should be discovered by some future Laennec, that such instrument would do more harm than good?

In the sentiments expressed in the following passage we perfectly coincide:

"The above are the principal changes which the biliary apparatus undergoes during life, and which can only be ascertained by the knife after death. But, it will be asked, 'can we not tell by the symptoms what is the organic change going on?' I venture to assert that we cannot. Since little can be learnt from external examination, in respect to the *kind* of structural disease in the liver, we have only the disorder of function, and its consequences on the constitution, to guide us; and I unhesitatingly aver, that disorder of function in the biliary apparatus is often more considerable where there is no change of structure, than where there is organic disease of great and irremediable magnitude. This is so much the case, that, when I find much functional disturbance in the biliary secretion, and much constitutional derangement resulting thence, I conclude (unless there be tangible enlargement) that the structure of the liver is unaffected in any material degree." (P. 34.)

We come now to explain to our readers the particular views which Dr. Johnson entertains concerning the disordered conditions of the digestive apparatus. Morbid sensibility of the nerves supplying the chylopoietic viscera is, in his view, the essential feature of this class of ailments, and he subdivides the subject into the two heads (or chapters) of Morbid Sensibility of the Stomach and Bowels attended *with* obvious disorder in the digestive organs, and the same *without* any obvious or well-marked symptom of such disorder. This arrangement corresponds very closely with the dyspepsia and hypochondriasis of most authors. In filling up the details, the author writes entirely from personal observation, and, we regret to learn, in a considerable degree also from personal suffering. This at once stamps a high value on the work, and, while perusing the painful catalogue of evils which spring from a disordered stomach, our sympathies with the author are assuaged by the reflection that, with the skill of the alchemist, he has converted the cup of sorrow into the potion of health, and has made individual suffering subservient to public advantage. Dr. Johnson expresses himself with very considerable power of language, and, though the passage is somewhat long, we are tempted to extract the following sketch of "biliary irritation acting on the mental faculties, through the medium of the intestinal nerves," as a very favourable specimen of his style.

"Severe as this paroxysm is, the patient may thank his stars that the poison vented its fury on the body instead of the mind. Where the intellectual faculties have been much harrassed, and the nervous system weakened, the morbid secretion acts in that direction, and little or no inconvenience is felt in the real seat of the enemy. The mind becomes suddenly overcast, as it were with a cloud; some dreadful imaginary evil seems impending, or some real evil, of trifling importance in itself, is quickly magnified into a terrific form, attended apparently with a train of disastrous consequences, from which the mental eye turns in dismay. The sufferer cannot keep in one position, but paces the room in agitation, giving vent to his fears in doleful soliloquies, or pouring forth his apprehensions in the ears of his friends. If he is from home when this fit comes on, he hastens back; but soon sets out again, in the vain hope of running from his own wretched feelings. If he happen to labour under any chronic complaint at the time, it is immediately converted into an incurable disease, and the distresses of a ruined and orphaned family rush upon his mind and heighten his agonies. He feels his pulse, and finds it intermitting; disease of the heart is threatened, and the doctor is summoned. If he ventures to go to bed, and falls into a slumber, he awakes in the midst of a frightful dream, and dares not again lay his head on the pillow. This state of misery may continue for twenty-four, thirty-six, or forty-eight hours; when a discharge of viscid, acrid bile, in a motion of horrible fetor, dissolves at once the spell by which the strongest mind may be bowed down to the earth, for a time, through the agency of a poisonous secretion on the intestinal nerves! I believe such a train of symptoms seldom obtains except where there has been a predisposition to morbid sensibility, occasioned by mental anxiety, vicissitudes of fortune, disappointments in business, failure of speculations, domestic afflictions, or some of those thousand moral ills which render both mind and body so susceptible of disorder. It is under the influence of such paroxysms as these, I am thoroughly convinced, that nine-tenths of those melancholy instances of suicide which shock the ears of the public take place." (P. 56-7.)

Dr. Johnson offers some very clear, and we think most satisfactory, criticisms upon the views of indigestion taken by Dr. Wilson Philip. After giving his reasons for distrusting Dr. Philip's ideas concerning tenderness of the epigastrium, he thus expresses himself regarding the peculiar hardness of the pulse noticed by that author.

"On this subject, I must take the liberty of saying that Dr. Philip appears to have refined to an excessive degree of minuteness. If a physician's whole sense was concentrated in the point of his forefinger, he would hardly be able to follow Dr. Philip in his diagnostic of hardness in a dyspeptic pulse. This hardness is often to be recognised only by 'a particular way' of feeling the pulse. 'If the pressure be gradually lessened till it comes to nothing, it often happens that a distinct hardness of the pulse is felt before the pulse wholly vanishes under the finger, when no hardness can be felt in the usual way of feeling it.' I appeal to the experience of every practitioner, whether such a refinement as the above can be entitled to much confidence in the examination of a phenomenon like the pulse, which varies with almost every emotion or thought that crosses the mind of a dyspeptic invalid. Is it to be assented to, that, by such a criterion as this, we shall be enabled to distinguish irritation from inflammation; or functional from organic disease? The fact is, that, in irritation of the stomach or bowels, the pulse is often as hard and as quick as in inflammation of those parts. The heart is so much under the influence of the stomach, in functional derangement of the latter organ, that no dependence can be placed on the state of the pulse, whether as regards hardness, frequency, or irregularity." (P. 65.)

To the importance and correctness of this last observation we are ready to offer our unqualified testimony. The chief peculiarities of Dr. Johnson's opinions concerning morbid sensibility of the stomach and bowels, are brought under the reader's notice when treating of the causes and management of hypochondriacal disorders. We may thus enumerate them:—Dr. Johnson's first position is, that our food and drink should never produce sensation in the stomach. This he considers as "one of the most fundamental views in pathology, and as leading to one of the most useful precepts in the art of preserving health."

"The moment we call forth conscious sensation in the stomach, whether that be of a pleasurable or a painful kind, we offer a violence to that organ, however slight may be the degree. Whenever the conscious sensibility of the stomach (or, indeed, of any other internal organ,) is excited by any thing we introduce into it, —by any thing generated in it,—or by any influence exercised on it through the medium of any other organ, we rouse one of nature's sentinels, who gives us warning that her salutary laws are violated, or on the point of being violated. Let us view the matter closer. We take an abstemious meal of plain food, without any stimulating drink. Is there any conscious sensation produced thereby in the stomach? I say, no. We feel a slight degree of pleasant sensation throughout the whole frame, especially if we have fasted for some time previously, but no distinct sensation in the stomach. There is not, there ought not to be, any conscious sensibility excited in this organ by the presence of food or drink, in a state of health; so true is the observation, that to feel that we have a stomach at all is no good sign." (P. 45.)

Farther on, he thus reiterates the principle—

"Any discomfort of body, any irritability or despondency of mind, succeeding food and drink, at the distance of an hour, a day, or even two or three days, may be regarded (other evident causes being absent) as a presumptive proof that the quantity has been too much, or the quality injurious." (P. 75.)

Another peculiarity in Dr. Johnson's views is, that hypochondriasis is, in general, independent of actual dyspepsia.

"I do maintain that, although hypochondriacal symptoms often attend indigestion, (as indeed I have abundantly shown,) yet indigestion is by no means essential to hypochondriasis. In two patients whom I am now attending, and who are perfect models of hypochondriacism, the appetite is good, the evacuations perfectly natural, and no pain, flatulence, or other symptom of indigestion in the stomach, is complained of. In both these instances, however, the hypochondriasis may, at pleasure, be exasperated or mitigated by free or by abstemious living; shewing that the nerves of the stomach and bowels are concerned in the mental phenomena." (P. 80.)

A little farther on (viz. page 82,) he states that "a morbid sensibility of the nerves of the stomach and bowels is the leading feature of hypochondriasis, and the cause of the varied and endless train of symptoms which develop themselves in the mind and in distant parts of the body. This may exist both with and without the usual symptoms of disordered digestion.

Dr. Johnson's observations on the causes of morbid sensibility of the stomach and bowels are, we think, particularly valuable. He lays stress on the following, and, in our opinion, with the greatest justice:—1, atmospheric variations; 2, errors in diet; 3, mental anxiety. On the important subject of the *moral* causes of stomach complaints, the author expresses himself in the following vigorous manner:

"In this country, where man's relations with the world around him are multiplied beyond all example in any other country, in consequence of the intensity of interest attached to politics, religion, commerce, literature, and the arts; where the temporal concerns of an immense proportion of the population are in a state of perpetual vacillation; where spiritual affairs excite great anxiety in the minds of many; and where speculative risks are daily run by all classes, from the disposers of empires in Leadenhall-street, down to the potatoe-merchant of Covent-garden,—it is really astonishing to observe the deleterious influence of these mental perturbations on the functions of the digestive organs. The operation of *physical* causes, numerous as these are, dwindles into complete insignificance, when compared with that of anxiety or tribulation of mind. These causes very often escape the investigation of the physician, unless he is very much on his guard. The patient is prodigal of description, as far as regards his corporeal feelings, and he is often very candid as to the physical causes which may be inquired after by the practitioner; but he seldom reveals (for obvious reasons) the real origin of the evil, when it is of a moral nature, unless it be dexterously drawn from him by artful cross-questioning." (P. 78.)

The most important feature in the work before us still, however, remains to be noticed. It regards the very interesting question of *treatment*, and as, in the sequel, we shall have to express our dissent from the author in some of his practical suggestions, we shall lay the subject before our readers in Dr. Johnson's own words. He says (page 89), "It is on the regulation of diet that our chief hopes of cure must rest. Instead of exhibiting purgatives day after day to carry off diseased secretions, we should lessen and simplify the food, in order to prevent the formation of these bad secretions." But the pith and marrow of the question will be best traced in the following remarks:

"I care not if the dyspeptic invalid begins with a pound of beef-steaks, and a bottle of port wine for his dinner. If he feel as comfortable at the end of two, four, six, eight, or twelve hours after this repast, as he did between breakfast and dinner of the preceding day, he had better continue his regimen, and throw physic to the dogs. But if, a few hours after his dinner, he feel a sense of distention in the stomach and bowels, or any of those symptoms of indigestion which have been pointed out; if he feel a languor of body, or a cloudiness of the mind; if he have a restless night; if he experience a depression of spirits or irritability of temper next morning, his repast has been too much, or improper in kind, and he must reduce and simplify till he come to that quantity and quality of food and drink for dinner, which will produce little or no alteration in his feelings, whether of exhilaration immediately after dinner, or of discomfort some hours after this meal. This is the criterion by which the patient must judge for himself. The scale of diet must be lowered and simplified down to water-gruel, if necessary; otherwise a cure can never be expected." (P. 90.)

Again—

"I have distinctly said that the invalid may eat and drink as much as he pleases, provided he experience no increase of his morbid feelings from food and drink, within the twenty-four succeeding hours. If he do feel an increase of these, the necessity of the restriction which I propose is self-evident, and, so far from being the imposition of a penance, it is, in reality, the removal of one. Let it be remembered that I am speaking of the dyspeptic stomach, and not of that which is in the enjoyment of all its healthy powers and of all its natural sensibilities. But the invalid may ask, 'Can I not have my ailments removed without abridging my appetites?' No! And the practitioner who undertakes the treatment under such conditions, betrays either a want of principle or a want of judgment." (P. 91.)

We have never suffered, like Dr. Johnson, from dyspeptic complaints, and therefore we are not such good judges of the matter as he is, but we doubt very much whether this *golden* rule, as he calls it, *of restraining the food to that quantity which produces no languor after eating, no unpleasant sensation of mind or body during digestion,* be in reality practicable upon a large scale. We suspect that, in many cases, the almost total withdrawing of nourishment which this plan leads to, would weaken the general system more than the author supposes. Water-gruel is, we allow, a sufficient diet for persons in a fever; but we are disposed to hesitate ere we assent to the following panegyric on the virtues of gruel in *chronic* ailments. "From four ounces of gruel every six hours, a patient will, under many states of indigestion, derive more nutriment and strength than from half a pound of animal food and a pint of wine." (P. 89.) Again he says, "Perhaps, of all species of food, this (gruel) is the least irritating, and, where a high degree of morbid sensibility prevails, it is often the only thing that can be borne." (P. 76.) And, for fear we should forget the fact, he tells us once more, (page 92,)

"The least irritating, and the most easily digested aliment, is unquestionably farinaceous food, at the head of which we may place good grit gruel. I have known many who could digest only this, without unpleasant sensations in the stomach or other part of the body. When such is the case, the nerves of the stomach are in a high degree of morbid sensibility, and great caution should be taken not to irritate them by attempts at more nutritious food. No person is in danger of starvation who can take a pint,—nay, only half a pint, of good gruel in the twenty-four hours. Arrow-root, sago, tapioca, rice, salep, are all in the same class; but few of them will bear repetition so well as gruel. A little sugar, and a tea-spoonful of brandy, in each cup of the gruel may be permitted, but the brandy may be safely dispensed with in general."

We cannot go into the minutiæ with which our author directs the diet of a dyspeptic invalid. We may state, however, that he is as much an enemy to wine, as he is to the solid delicacies of the table. "My firm conviction is, that, in respect to drink, water is the best; and, till the habit of water-drinking can be acquired, a dilute mixture of brandy and water is the next best beverage." (P. 94.)

We have, then, the strongest assurances given us that this rigid system of diet is not the creature of speculation, but the dictate of experience. We are even told that "those who have persevered in it, and reaped its fruits, will hardly be induced to change it afterwards, however strongly they may be tempted by the luxuries of the table, or the seductions of convivial society."

Dr. Johnson, however, does not discard the use of medicine. He acknowledges that benefit may occasionally be derived from it, especially with the view of expelling and correcting the morbid secretions which are poured into the alimentary canal. The following remarks are excellent:

"Infinite mischief, as I have stated before, is daily occasioned by the indiscriminate employment of purgative medicine, in dyspeptic complaints. Bad secretions may be thus removed, but their reproduction will never be thus prevented. It is by withdrawing the sources of irritation, and gradually improving the functions of the liver, the stomach, and the intestinal canal, that the formation of morbid secretions can be arrested. Purgation, therefore, should be rarely employed. It may be proper, just at the beginning, to clear the alimentary canal of all its lurking contents; but, after this, I do maintain that the main object is to produce but one evacuation daily, and that of a solid rather than a liquid consistence. If practitioners knew the misery that is often produced by an irritating cathartic medicine in dyspeptic and hypochondriacal complaints, in this country, they would be more sparing than they are of their calomel at night and black draught in the morning." (P. 97.)

A variety of formulæ are then given, applicable as habitual aperients. They are all very complicated, the author believing that in this manner a more complete effect is produced along the line of the digestive apparatus. Where morbid sensibility prevails to any considerable degree, we are advised to join hyoscyamus, or some other gentle anodyne, to the aperient. The efficacy of counter-irritants, especially of a plaster containing the tartarised antimony, of anodynes, and of the tepid bath, is then briefly touched upon. Some valuable observations follow on the employment of bitters and tonics in cases of indigestion, and morbid sensibility of the stomach and bowels. The sulphate of quina, in half-grain doses, is spoken of as very efficacious in cleaning the tongue, improving the appetite, and imparting tone and tranquillity both to mind and body.

Dr. Johnson extols the nitrate of silver as a very valuable remedy in the removal of morbid sensibility of the primæ viæ. He does not, indeed, hold it out as a specific, but contends that it may be often resorted to with a fair prospect of benefit. He considers that the good effects occasionally experienced from this medicine in epilepsy, depends on its power of lessening the sensibility of nerves. It appears to us, however, that the author here writes from theory rather than actual practice. He mentions cases wherein he expects beneficial results, and where the patients are on the point of being cured. The medicine may be given in the dose of half a grain, at bedtime, in combination with any bitter or aperient extract.

The last subject which occupies our author's pen is *travelling*, and its moral and physical effects. On this topic Dr. Johnson is particularly eloquent. In the autumn of 1823, he made the grand tour of Europe, for the sole purpose of health, and the result appears to have exceeded his most sanguine expectations. We have not space for the author's detailed exposition of the effects of travel upon the mental and bodily functions, but we must find room for the following summing-up:

"These unequivocally good effects of travelling on the digestive organs, account satisfactorily for the various other beneficial influences on the constitution at large. Hence dyspepsia, and the thousand wretched sensations and nervous affections thereon dependent, vanish before persevering exercise in travelling, and new life is imparted to the whole system, mental and corporeal. In short, I am quite positive that the most inveterate dyspepsia (where no organic disease has taken place) would be completely removed, with all its multiform sympathetic torments, by a journey of two thousand miles through Switzerland and Germany, conducted on the principle of combining active with passive exercise in the open air, in such proportions as would suit the individual constitution and the previous habits of life." (P. 124.)

This brings us to the conclusion of the volume,—a volume, we repeat, small in size, but rich in matter, from the perusal of which every reader will derive instruction. The author is laudably content to give us the result only of his personal feelings, and of his reflections upon them; and the extracts which we have given sufficiently attest the value of his contribution to the stock of medical facts. The essay is written throughout in a pleasing, unaffected style. In one passage, indeed, (page 77,) there is an attempt at rhetorical flourish. Speaking of the moral causes of disordered stomach, we are told, " that there is but one path along which they travel from the organ of thought, but the number of these airy sprites, and the velocity with which they glide along the silvery pneumo-gastric conductors, baffle all calculation." This little blemish, if so we may term it, is however amply compensated by many vigorous passages, and by the general tone of good sense and sound reasoning which pervades and characterises the work.

---

*On Galvanism; with Observations on its Chymical Properties and Medical Efficacy in Chronic Diseases, with Practical Illustrations; also, Remarks on some auxiliary Remedies. With Plates.* By M. La Beaume, Medical Galvanist and Surgeon-Electrician, F.L.S. &c.—12mo. pp. 271. Highley, London, 1826.

This volume is obviously intended more for the public than the profession, as it is prefaced by a plate of the " thoracic and abdominal viscera," for those " intelligent readers who may be unacquainted with the anatomy and physiology" of those parts. The author is anxious to separate himself from the " mere mechanical operators of electricity." He " attacks disease at its source by one of the most powerful medical agents that has ever been employed in the curative treatment of human maladies."

The accidental discovery of animal or metallic electricity may not be known to all our readers, and is sufficiently curious to be briefly mentioned. It was first observed by the wife of Galvani, anatomical professor at Bologna, in 1791.

" Some frogs, recently skinned for cooking, were casually lying on a table near an electrical machine in the laboratory of the professor. After the apparatus was put into action, an attendant unintentionally touched with the point of a scalpel the crural nerves of the frog, that was nearest to the prime conductor, when the muscles of the limb were instantly thrown into strong convulsions. This remarkable circumstance the wife communicated to her husband, who repeated the experiment with the same result; which appeared to confirm the hypothesis he had previously formed, that muscular motion depended on electricity. This ingenious man subsequently made many experiments on frogs, from which he concluded that there is in all animals an inherent electricity, capable of exciting muscular motion on the application of dissimilar metals to different parts of the body. Hence the science of animal or metallic electricity, as well as the fluid, is called after his name. Soon after Galvani had made known his discoveries, several philosophers, among whom were Valli, Fowler, Fabroni, and others, prosecuted their inquiries into this new phenomenon. Their researches confirmed the important fact, that galvanism possesses a peculiar power in exciting the nerves. Sultzer, a German writer, was the first who noticed the effects which two dissimilar metals have on the tongue, when brought into contact, and which produce a subacid taste. This discovery evinced the influence of the galvanic principle on the gustatory nerves; and, some time after, its effect on the optic nerves, in producing a flash of light, was observed by Fowler. Similar experiments were also made by others with the same results; and this application of two dissimilar metals constitutes the simple galvanic power." (P. 3.)

It may be easily conceived that the spirit of inquiry, and zeal for improvement, led many other individuals to prosecute their investigations upon the subject. Fourcroy, Vauquelin, Tiomsdorf, Biot, Cuvier, &c. &c. have, in foreign countries, contributed much to the advancement of the science of galvanism. In England we have not been idle. To Mr. Cruickshanks, of Woolwich, we are indebted for the galvanic battery, " by which Dr. Henry decomposed the sulphuric and nitric acids, ammonia, &c., and by which Sir Humphry Davy achieved his greatest chemical exploits."

Electricity and galvanism are not unfrequently confounded. The differences between them are—

" First, in their development; secondly, in their state; thirdly, in their action; and, fourthly, in their effects.

" First: The development of the electric fluid is obtained by *mechanical* friction; that of the galvanic fluid by a *chemical* action.

" Secondly: The electric fluid exists in a highly elastic state; and its particles are strongly repulsive of each other, and not disposed to form a permanent union with other bodies. The galvanic fluid, on the contrary, has the strongest tendency to form new combinations; which tendency, as Sir Humphry Davy observes, is so powerful as to counteract some of the strongest chemical affinities. Electricity may be compared to an agent in a state of great dilution or expansion; galvanism to an agent of great concentration and intensity. The former has been compared to the flame of a candle; the latter to the flame of a blow-pipe.

" Thirdly: The electric fluid, in its immediate action, causes great commotion in its passage from one body to another, its particles being mutually repulsive; whereas galvanism enters more readily into bodies, and without creating any great commotion, on account of its tendency to form new combinations.

" Fourthly: The electric fluid is more powerful in its immediate, than in its ultimate effects: the galvanic fluid, on the contrary, is more powerful in its ultimate than in its immediate effects.

Hence is deduced the superiority of the medicinal energy of galvanism; and from this important fact has arisen the idea, that where electricity ends galvanism begins." (P. 15.)

M. La Beaume has dedicated many years to acquiring a knowledge of electricity and galvanism, and may be pardoned for a little enthusiasm in his favourite study. It is probably true that too little attention is paid by practitioners in general to the powerful effects that may be produced by those agents, whilst it is equally certain that those who have especially devoted themselves to those subjects have not under-rated their curative powers.

We should be gratified to learn whether asthma, for instance, is "relieved almost uniformly"* by the galvanic influence, in the present day, as it was reported to be some ten years ago in a provincial hospital; and whether there is more unity of opinion upon the subject, amongst those who have an opportunity of witnessing the experiments, than existed at the time of the publication of the cases to which we refer.

M. La Beaume thus states his opinion of the general application and effects of galvanism to disease.

"First: Galvanism is not at all applicable to acute or inflammatory diseases, or to those disorders which are occasioned or perpetuated by a high degree of arterial or nervous excitement.

"Secondly: Galvanism is very beneficial as a topical remedy in some local diseases, which are not dependent on a constitutional derangement of the system, nor occasioned by an organic or structural change of the parts.

"Thirdly: Galvanism is often serviceable as a palliative means, affording the greatest relief in organic diseases; but it is most effectual in the cure of functional disorders, and in local affections connected with the general health, or altogether dependent on it.

"The medical properties of galvanism are stimulant, derivative, resolutive, and deobstruent. Its remedial power, as a natural excitant of the vital forces, vastly exceeds its energy as a local application; for it has not only a most powerful influence on the nerves and muscles, but also on the arterial and vascular systems; for it increases the action of the heart and arteries, and consequently the fulness or frequency of the pulse, and sometimes both; it also equalises animal heat, restores the balance of circulation, and exhilarates the spirits. Its beneficial effects on the glandular system, (which is at present little known to medical men,) is astonishingly great; for it promotes healthy secretions of the liver, kidneys, and other glands, as well as those of the stomach, bowels, and the skin. It removes spasmodic affections, and allays the morbid irritation of the nervous system, by its tonic and invigorating

* The quotation is not from M. La Beaume.—Reviewer.

influence on the chylopoietic organs. Thus it not only restores corporeal strength, but also nervous, sensorial, and intellectual power. Galvanism is especially effectual in removing biliary obstructions, and in curing those chronic disorders of the liver occasioned by a residence in hot climates, as well as torpidity of that viscus, induced by sedentary occupations, or intemperate habits. In these cases it has been found an efficient and beneficial substitute for mercurial medicines, as its action is exceedingly mild and perfectly safe, and does not entail those ruinous and distressing effects on the constitution, which generally follow a course of mercury. Galvanism is also the more desirable, because, during its administration, it does not require confinement to the house, or any other inconvenient restraint: on the contrary, air, exercise, and a generous diet, are found materially to aid its curative process. Though I am fully authorised by my experience to make these observations, yet I do not assert that galvanism is an infallible remedy for every case of disease to which it is applicable, nor that the occasional use of gentle medicines is to be superseded. But I most positively affirm, that such is its sanative powers in the deranged functions of the digestive organs, that it has, in numerous instances, effected the most extraordinary cures after the failure of every other internal and external means, which had been most judiciously prescribed and perseveringly used, under the direction and superintendence of the ablest practitioners. As galvanism is a natural excitant, its effect on the human frame is not like that of ardent spirits, and mineral stimulants or tonics, which are generally temporary. The recovery which has been obtained by the galvanic course has, in the greatest number of cases, been lasting. These facts, which I boldly state, can be borne out by the testimony of a number of respectable individuals of unquestionable veracity, in different classes of society, who have experienced the permanently beneficial effects of galvanic agency, in the cure of obstinate and complicated diseases, which has baffled all other efforts of the best medical treatment." (P. 25—9.)

The different forms in which the author has administered galvanism are—by a gentle stream, an interrupted current, or vibratory impulses, and not by shocks. There is a manifest dissimilarity between the sensations and effects produced by each of these forms. It rarely happens that any disagreeable sensation is produced, unless from the fearful apprehensions of the timid patient, or the morbid susceptibility of his nervous system. In the course "of the last nine years, I have had (says the author,) about eight hundred cases of stomach, liver, and bowel complaints, accompanied by many distressing local affections, which have been relieved and cured in the proportion of eight cases out of ten." The success is great.

M. La Beaume has "not had many cases of diseased spleen, but in the few that have occurred galvanism has been very useful." Even encysted dropsy of the ovarium, "by the alternation of electricity and galvanism for several months," was entirely removed.

We cannot follow the author through his enumeration of a great proportion of the diseases to which the human body is subject, and in which galvanism is said to have been employed with more or less effect. We would suggest to him the difficulty of determining, particularly in cases of dyspepsia and hepatic derangements, how much benefit he ought to have placed to the account of galvanism, and how much to the "occasional medicines and dietetic rules" he prescribed.

We cannot offer any comments upon the cases with which the book abounds. They are generally dismissed in a few lines, and are much too indefinitely stated to be put to the test of criticism.

We have no doubt M. La Beaume is well instructed in the science upon which he writes, but we are rather surprised that he should think it necessary to assert that he "never made galvanism a stepping-stone to notoriety." He need not be ashamed of any notoriety he may acquire in the fair and liberal prosecution of a useful science.

---

*An Essay on the Use of the Atropa Belladonna, or Solanum Lethale, and the Solanum Hortense; with Practical Observations on their Effects in the Cure of Scirrhus, Cancer, Stricture, and various other Complaints.* Dedicated, by permission, to His Royal Highness the Duke of Clarence. By POWELL CHARLES BLACKETT, Member of the Royal College of Surgeons in London, Surgeon in the Royal Navy, and Surgeon Extraordinary to His Royal Highness the Duke of Clarence, &c. &c. &c. —8vo. pp. 68. Callow and Wilson, London, 1826.

THE object of this Essay is to call the attention of the profession to the virtues of the Belladonna. That this plant is a narcotic of great power, is universally known and acknowledged, and its application to certain purposes (as effecting dilatation of the pupil) is very generally adopted. Mr. BLACKETT, however, is not contented with this, nor with the occasional recourse which is had to the remedy for the fulfilment of those indications, which we usually attempt by some milder anodyne; and steps forward to vindicate the claims of belladonna to a higher rank in the class of drugs, and to a more general adoption in medical practice. This medicine, he says, "should be given in doses varying according to the indications that lead to its adoption. It should be prescribed in small and repeated doses, to operate gradually through the medium of the circulation. In this manner exhibited to influence the kidneys, and prove *diuretic;* the intestines, and prove *purgative;* the skin, and prove *sudorific;* the nerves, and prove *sedative;* the absorbents, and prove the *agent of absorption*; and by these means become beneficial in the various complaints in which it is employed." If, indeed, the belladonna be capable of producing all those effects which we have marked by placing them in italics, it must be acknowledged to possess medicinal agencies of the highest rank; but, notwithstanding our free admission of those powers as a narcotic to which we have alluded, and our full conviction of Mr. Blackett's perfectly good faith, yet we cannot but look upon such varied and multifarious properties as resulting from that favouritism with which men are prone to view the merits of what they have themselves been induced to patronise. Nor is this opinion lessened on finding (at page 46,) that the author thinks it "a remedy of the utmost importance in schirrus, in cancer, and in cancerous ulcers, &c., and, according to the best historical authorities on this subject, we find that these diseases have been cured, as well as palliated, by it in their most distressing stages." We must dissent from the accuracy of this assertion. Mr. Blackett must be quite aware that when Stoerk, of Vienna, took all the narcotics, and particularly cicuta, under his especial protection, that they were all (and belladonna among the rest) fully and fairly tried—and found wanting. That schirrus and cancer may be *palliated* by this or any other powerful narcotic, we admit: that they have ever been *cured* by them, we doubt. "A tumor, (says the author,) being at first a hard glandular swelling, without inflammation, is called schirrus." (P. 47.) If, indeed, every tumor having these characters is to be regarded as *schirrus,* then very possibly belladonna may cure this disease; but so will almost any thing else; for, in truth, the recovery will often take place without any remedies being employed. But it is quite obvious that such tumors are often wholly unconnected with any cancerous taint: while with regard to true schirrus, as it will frequently remain for many years quiescent till roused into activity by some unfortunate circumstance, so, when belladonna happens to have been administered in such a case, the indolence of the tumor will naturally be attributed to its controlling influence.

Mr. Blackett states that he has seen belladonna of use in seven cases of tumor; but of these only two are said to have been of a cancerous nature; whereof the one proved fatal, and the other is not described, but is merely called a "cancerous ulcer:" it runs as follows.

"Case II.—W. W. aged forty-five, residing in Southampton-row, applied to me in 1823: she had a cancerous ulcer of the left mamma, about the size of a crown. The belladonna was taken internally, and applied externally, varied in doses and applications according to the symptoms, &c., and, in about nine or ten weeks after the first use and application of the remedy, she recovered." (P. 50.)

We are of opinion, then, that Mr. Blackett has not confirmed, by any sufficient proof, his position with regard to the use of belladonna in schirrous affections. At the same time, we have no doubt that it is a very important remedy in various painful and nervous diseases, as described in the pamphlet before us; illustrations of which may be found in papers by the author, in preceding Numbers of this Journal.

---

*The Use of the Chlorate of Soda, and the Chlorate of Lime.* By A. G. Labarraque, Pharmacien of Paris, Member of the Society of Medicine, of the Free Society of Pharmaciens, Resident Assistant Member of the Royal Academy of Medicine, &c. Translated by James Scott, Surgeon.—8vo. pp. 36. Highley, London, 1826.

The Chlorates of Lime and of Soda have lately been extensively used in Paris, as disinfecting agents, and with the most satisfactory results. The effects of the former, particularly, are of the most decided kind in destroying the offensive smell of putrifying animal substances. During the very hot weather of last summer, we witnessed some experiments which were tried with this preparation on a quantity of animal matter which had been purposely set aside to rot. The stench was intolerable: a small quantity of chlorate of lime in solution was poured over it, with the effect of immediately removing the smell altogether. The utility of such an agent in conducting the dissection of bodies far advanced in putrefaction, and indeed under a great variety of other circumstances, is too obvious to require that we should insist upon it. We shall, therefore, content ourselves with having brought the subject under the notice of our readers, and by giving such extracts from the pamphlet of M. Labarraque as are necessary to make the method of its application intelligible.

"*Disinterment and inspection of dead bodies.*—Before approaching a dead body in a state of putrefaction, it is necessary to procure a vessel, into which a quantity of water (twenty-four litres*) is to be put, and into this about eighteen ounces (demi-kilogramme) of the chlorate of lime is to be poured, and the mixture well agitated.

"A piece of linen cloth is to be put into the vessel containing the fluid, in such a manner that it can be withdrawn easily and quickly; for this purpose two persons open the cloth, and, holding it by the corners, immerse it in the liquid, which is to be placed close to the putrid body, and at the same instant the wet cloth is withdrawn from the vessel and spread on the subject, and soon after the putrid odour ceases.

"If there should be any blood or other fluid issuing from the body, a glass or two of the chlorated water should be poured on it. It should then be rubbed with a brush, and the fetid odour will disappear.

"This operation ought not always to be prosecuted exactly in the manner above described, as it is necessary, in cases where the surface of the body is uncleanly, that it should be cleansed as thoroughly as possible before the disinfecting process is commenced.

"If the taint has extended into the surrounding apartments, galleries, staircases, &c. the places infected should be sprinkled with one or two glasses of the liquid, and the stench will cease.

"Care should be taken that the cloth covering the body be constantly sprinkled with the liquid contained in the vessel; thus the reproduction of the fetid odour is prevented." (P. 8.)

"These two chlorates (of lime and soda) are equally proper for checking putrefaction, yet have not both the same secondary properties. I will explain myself. In the act of disinfecting putrid animal matter, the chlorate passes into a state of hydro-chlorate, and the hydro-chlorate of lime, having the property of absorbing humidity, combines with the moisture of the disinfected body. Now, one of the conditions of putrefaction being moisture, it follows that, when once the disinfecting action is in operation, the chlorate, after a longer or shorter time, according to the quantity, changes its state, and furnishes materials for reproducing the fetidness. On the contrary, the chlorate of soda, in passing to the state of an hydro-chlorate, produces the formation of a very dry salt, that absorbs the moisture, which is the principle of putrefaction. This is what I term a secondary property. The chlorate of soda, therefore, when used for disinfection, prevents, at all times, the reproduction of putrefaction. It is particularly suitable for application to sores of bad character, from the property which it possesses of detaching the disorganised tissue from that which preserves its vital qualities; whilst the chlorate of lime, if it is well saturated, can be used only for simple disinfection, such as in the

* About six gallons.

exhumation of bodies which are to be immediately examined. It is also suitable for the disinfection of the bodies lying at the Morgue, because the sprinklings of the chlorated water are there frequently renewed. The chlorate of lime is eligible for disinfecting water-closets, and for this purpose gentle sprinklings only are necessary, renewed from time to time as required." (P. 28.)

---

*Materia Indica; or some Account of those Articles which are employed by the Hindoos, and other Eastern Nations, in their Medicine, Arts, and Agriculture: comprising also a Formulæ, with Practical Observations, Names of Diseases in various Eastern Languages, and a copious List of Oriental Books immediately connected with general Science, &c. &c.* By WHITELAW AINSLIE, M.D. M.R.A.S. late of the Medical Staff of Southern India.—In two Volumes, 8vo. Longman and Co. London, 1826.

THIS work consists of a list of the different articles employed by the natives of Hindostan in medicine, as well as in the arts, and is an enlarged edition (with a new name) of one published in India in 1813. The first volume contains such of the drugs used in Europe as are found in Eastern countries; the second, those found in Eastern countries, but not used in Europe. To make this compilation must have been a work of prodigious labour, and it is impossible to imagine any thing less inviting to the general reader than the result. Indeed, these volumes can never be looked upon otherwise than as an authority to which we may refer for information respecting any particular substance. In this way they will be of use to those who have occasion to practise in Oriental countries, and probably to none other. The second volume likewise contains a list of books in various Eastern languages, connected with medicine and other sciences.

After the name of each article in various Eastern languages, a short account of it is appended, thus—

"ADDATINAPALAY.—*Gadíday gudda purra* (Tel.) *Floral-leaved birthwort. Cattrábunghá* (Sans.) ARISTOLOCHIA BRACTEATA (Retz.)

"Cl. and Ord. Gynandria Hexandria. Nat. Ord. Sarmentaceæ. *Beblatterte Osterluzey* (Nom. Triv. Willd.)

"Of the essential character, Willdenow says, '*Cal.* 0; *cor.* 1-petala, lingulata, basi ventricosa; *caps.* 6-locularis, polysperma infera" (Spec. Plant. iv. 1609.)

"This species of birthwort, which may be seen in the botanical garden of Calcutta, appears to have been first noticed by Kœnig, in the neighbourhood of Madras; it usually 'grows to the height of about four or five feet, with a flexuose, striated stem; the leaves, which are of a pale green, are obtuse, heart-shaped, with wavy edges, and about an inch and a half long, and nearly as broad; the flowers are solitary; and the bractes cordate petioled.' The plant has the bitterness which distinguishes many of its congeners. An infusion of the dried leaves is given by the native practitioners as an anthelmintic; the medium dose about ℥ij. twice daily. When fresh bruised, and mixed with castor-oil, they are considered as a valuable external remedy in obstinate psora. Dr. Fleming informs us that the root of the Aristolochia Indica is supposed, by the Hindoos of Upper India, to possess emmenagogue and antarthritic virtues; and, from its bitterness, he thinks it may be useful in dyspepsia. The plant is *isarmel* in Hindoostanie; *dulago-vila* in Tellingoo; *cay-khoaica* in Chinese; *hari* in Sanscrit; and *sat sanda* in Cyngalese. The Aristolochia Odoratissima, a native of the West Indies, Lunan says, is, as a bitter and alexipharmic, a most valuable medicine, being powerfully tonic and stomachic; he adds, that the roots and seeds cure the bites of snakes, and make the best bitter wine in the world!" (P. 4, vol. ii.)

---

*A New Supplement to the Pharmacopœias of London, Edinburgh, Dublin, and Paris; forming a complete Dispensatory and Conspectus; including the new French Medicines, as well as Herbs, Drugs, Compounds, Veterinary Drugs, Patent Medicines, Perfumery, Paints, Varnishes, and similar Articles kept in the Shops; with their Composition, Uses, Doses, and Adulterations; being a general Book of Formulæ for daily Reference in the Laboratory, and at the Counter.* By JAMES RENNIE, A.M. Surgeon; Lecturer on Chemistry, Natural History, and Philosophy, London; Editor of the Quarterly Journal of Foreign and British Medicine; Author of a Conspectus of Prescriptions in Medicine, Surgery, and Midwifery, the Pharmacopœia Imperialis, &c.—8vo. pp. 490. Baldwin, Cradock, and Joy, London, 1826.

THIS is a dictionary of medicines, regular and irregular; of simples and compounds; of drugs, nostrums, and perfumeries; herbs and chemicals; sauces and other poisons; liqueurs, wines, cordials, waters, spirits, essences, oils, soaps, dyes, jellies, pickles, preserves, and, in short, of "every thing in the world" relating to all that the truly omnivorous animal, man, puts into his stomach, for preserving health, or for destroying it; or which he applies externally for the convenience, comfort, ornament, or disfigurement of his person. These articles are arranged alphabetically, by which the reference is rendered very simple; while under each head is a short, but distinct, account of the substance.

To give a more extended notice of the work would be as impracticable as to analyse JOHNSON's Dictionary: our readers, therefore, must be contented with our opinion, that much pains seem to have been bestowed upon the compilation, and that it will be found very generally useful.

---

*Lexicon Pharmacopœlium, or a Pharmacopœial Dictionary; containing the London Pharmacopœia of* 1824, *in Latin and English; the Chemical Decompositions; a Description of the Simples and Compounds of the Pharmacopœias of London, Edinburgh, and Dublin, with their Properties, Use, and Doses; and in which all former Names are referred to the present Pharmaceutical Nomenclature. To the whole is annexed an English Index of the Technical and Domestic Terms relative to Medicines; a Table shewing at one view the Common and Linnæan Names;* Dr. CULLEN's *and* Mr. JOHN MURRAY's *Arrangement of the Materia Medica; a Dictionary of Operative Medicine; and, lastly, two serviceable Vocabularies for Translating Physician's Prescriptions and the Pharmacopœias. Designed expressly for the Use of Students.* By THOMAS CASTLE, Member of the Physical Society, Guy's Hospital.—18mo. pp. 327. Cox and Son, London, 1826.

THE contents of this little volume are so fully described in the title-page, as to require very little additional notice from us. Like the preceding volume, it is a dictionary of medicines and pharmaceutical terms, but is more limited in its object, and less complete in itself. It is, however, more condensed and portable: it is "expressly designed for the use of students," and the manner of its execution, and the tables and vocabularies it contains, will render it of very great assistance to the medical pupil.

